# Editorial: Cancer biomarkers: molecular insights into diagnosis, prognosis, and risk prediction

**DOI:** 10.3389/fmolb.2025.1636721

**Published:** 2025-06-06

**Authors:** Matteo Becatti, Shaoquan Zheng, Giuseppe Bronte

**Affiliations:** ^1^ Department of Experimental and Clinical Biomedical Sciences “Mario Serio”, University of Firenze, Firenze, Italy; ^2^ Breast Disease Center, The First Affiliated Hospital, Sun Yat-sen University, Guangzhou, China; ^3^ Department of Translational Medicine, University of Ferrara, Ferrara, Italy; ^4^ Department of Oncology, University Hospital of Ferrara, Ferrara, Italy

**Keywords:** cancer, biomarker, diagnosis, prognosis, risk prediction, tumor

Cancer biomarkers represent a critical frontier in precision oncology, enabling more refined stratification of patients, earlier detection, improved therapeutic targeting, and more accurate prognostic estimation. This Research Topic assembles a Research Topic of 14 diverse yet thematically converging articles that reflect the state-of-the-art and evolving paradigms in biomarker science across a variety of tumor types.

Several studies in this Research Topic identify gene expression drivers of tumor progression and therapy resistance, such as DAP3 in hepatocellular carcinoma, HJURP in gastric cancer, and ANTXR1 in bladder cancer. DAP3 was shown to promote tumor proliferation, invasion, and resistance to apoptosis, with a predictive model constructed based on its expression (Yuan et al.). HJURP was found to modulate chemoresistance via the MYC/TOP2A axis in gastric cancer, supporting its candidacy as a dual biomarker and therapeutic target (Liu et al.). ANTXR1 expression correlated with stromal scores and poor prognosis in bladder cancer, positioning it as both a prognostic marker and a candidate for targeted therapy (Franco et al.).

Other contributions focus on prognostic and predictive biomarker models. A meta-analysis of over 9,000 patients demonstrated that a high glucose-to-lymphocyte ratio (GLR) is a negative prognostic factor across various solid tumors, especially hepatocellular, breast, and pancreatic cancers (Li et al.). In esophageal squamous cell carcinoma, machine learning identified ATF2, ALOXE3, and SLC27A5 as predictive of response to chemoradiotherapy and overall survival, integrating gene expression, immune profiles, and drug sensitivity data (Cui et al.). Additionally, an epidemiological modeling study on lung cancer burden in BRICS nations revealed escalating trends in China and India, underscoring the importance of integrating biomarker strategies into public health policies (Wang et al.).

Innovations in diagnostic approaches also feature prominently. In colorectal cancer, immunolipid magnetic beads targeting EpCAM and vimentin enabled sensitive and specific capture of circulating tumor cells (CTCs), with a high concordance to tissue-derived mutation profiles (Deng et al.). Plasma autoantibodies to COPT1 showed potential as early diagnostic biomarkers for non-small cell lung cancer (NSCLC), with combining IgG and IgM achieving an AUC of 0.784, further enhanced by CEA inclusion (Cao et al.). In rectal carcinoma, diffusion-weighted MRI and ADC mapping distinguished malignancy from radiation-induced proctitis and normal tissue with near-perfect sensitivity and specificity, though not linked to prognosis (Šarošković et al.).

The immune microenvironment and its modulation through intrinsic tumor pathways are also explored. One study evaluated cGAS/STING pathway activation across 15 checkpoint inhibitor-treated cohorts (Chen et al.). While STING activation correlated with improved survival in some datasets (e.g., melanoma and bladder cancer), it was paradoxically associated with poor outcomes in others (e.g., renal carcinoma), suggesting tumor-type-specific immune dynamics. This is echoed by work on circMIB1 in glioma, which functions as a competing endogenous RNA sponging miR-1290, thereby upregulating tumor suppressor genes involved in apoptosis and circadian rhythm regulation (Chen et al.). These insights contribute to our understanding of non-coding RNAs in modulating oncogenic pathways and immunogenicity.

Additional complexity is addressed in rare or diagnostically ambiguous tumors. A bioinformatic study identified overlapping gene signatures between acute myocardial infarction and non-small cell lung cancer (NSCLC). COL1A1 and PLAU were identified as key hub genes with diagnostic value. Repurposable drugs, such as zoledronic acid, emerged for connection with hub genes. These findings supported the usefulness of the protein-protein interaction network analysis to identify hub genes (Zheng et al.). A case report detailed the misdiagnosis of pulmonary Ewing sarcoma as NSCLC, corrected through molecular pathology revealing an EWSR1FLI1 fusion gene (Carter et al.). This led to a treatment shift to VDC + I.E., chemotherapy, surgery, and radiotherapy with excellent response, highlighting the vital role of molecular diagnostics and reference pathology centers. A comprehensive review article synthesized established and emerging biomarkers in colorectal cancer, including KRAS, BRAF, MSI, HER-2, RET, and ctDNA, providing a framework for integrating biomarkers into therapeutic algorithms and clinical trial design (Zhang et al.).

Taken together, these studies underscore the multifaceted role of biomarkers in guiding diagnosis, prognosis, and decision for personalized therapy. They also highlight persistent challenges, including biomarker standardization, cross-platform validation, and accessibility in clinical settings. Yet, the collective insights affirm that the integration of omics data, computational biology, and translational research is reshaping oncology toward a more precise and patient-centered practice. This Research Topic demonstrates that continued interdisciplinary collaboration is key to unlocking the full potential of biomarker-driven oncology.

